# Patients Awaiting Surgical Repair for Large Abdominal Aortic Aneurysms Can Exercise at Moderate to Hard Intensities with a Low Risk of Adverse Events

**DOI:** 10.3389/fphys.2016.00684

**Published:** 2017-01-09

**Authors:** Matthew Weston, Alan M. Batterham, Garry A. Tew, Elke Kothmann, Karen Kerr, Shah Nawaz, David Yates, Gerard Danjoux

**Affiliations:** ^1^Sport and Exercise Subject Group, School of Social Sciences, Business and Law, Teesside UniversityMiddlesbrough, UK; ^2^Health and Social Care Institute, Teesside UniversityMiddlesbrough, UK; ^3^Department of Sport, Exercise and Rehabilitation, Northumbria UniversityNewcastle, UK; ^4^Department of Academic Anaesthesia, NHS Foundation Trust, South Tees HospitalsMiddlesbrough, UK; ^5^Department of Anaesthesia, Sheffield Teaching Hospitals Foundation Trust, Northern General HospitalSheffield, UK; ^6^Sheffield Vascular Institute, Northern General HospitalSheffield, UK; ^7^Department of Anaesthesia, York HospitalYork, UK

**Keywords:** pre-habilitation, HIT, intervention fidelity, training monitoring, safety

## Abstract

**Purpose:** Intervention fidelity refers to the extent an experimental manipulation has been implemented as intended. Our aim was to evaluate the fidelity of high-intensity interval training (HIT) in patients awaiting repair of large abdominal aortic aneurysms.

**Methods:** Following a baseline cardiopulmonary exercise test, 27 participants performed a hospital-based, supervised HIT intervention in the 4 weeks preceding surgery. The intervention was performed thrice weekly on a cycle ergometer and involved either 8 × 2-min intervals, each interspersed by 2-min recovery periods, or 4 × 4-min intervals interspersed with 4-min recovery periods. When surgery was delayed, participants undertook one maintenance HIT session per week until surgery. Session one power output was set to baseline anaerobic threshold power output and then increased on subsequent sessions until ratings of perceived exertion (RPE; Borg CR-10) for the legs (RPE-L) and sense of breathlessness/ chest (RPE-C) were hard (5) to very hard (7) at the end of each interval. For safety, power output was maintained or reduced if systolic blood pressure exceeded 180 mm Hg or heart rate exceeded 95% of maximum.

**Results:** Overall session attendance across the 4-week HIT intervention was 74%. Seventeen participants met our compliance criteria of ≥75% of intervention sessions and all maintenance sessions. When compared to non-compliance, compliant participants had higher fitness, performed more HIT sessions and were able to exercise at higher exercise intensities with a lower proportion of exercise safety breaches. In the 17 compliant participants, the proportion of repetitions meeting the HIT criterion was 30% (RPE-L) and 16% (RPE-C). Mean repetition intensity was 4.1 ± 2.0 Arbitrary Units [AU] (RPE-L) and 3.5 ± 1.9 AU (RPE-C) with a within-subject variability of ±1.4 AU and ±1.6 AU, respectively. We observed higher RPE scores (~0.5 AU) following 2-min intervals when compared to 4-min intervals and exercise power output increased 23% across the 4-week HIT intervention. One participant experienced an adverse event but were still able to complete their remaining exercise sessions.

**Conclusions:** Despite an inconsistent and lower than prescribed intensity, it is possible to exercise this high-risk patient population at moderate to hard intensities with a low risk of adverse events.

**Clinical Trial Registration:** http://www.isrctn.com/, registration number ISRCTN09433624.

## Introduction

Cardiorespiratory fitness is associated with post-operative outcome. Less fit patients have a higher incidence of morbidity and mortality (Snowden et al., 2013) whereas patients with adequate cardiorespiratory fitness are able to meet the increased physiological demands that accompany major surgery (Tew et al., 2014). Specifically, major surgery is associated with a variety of cardiopulmonary, neuroendocrine and metabolic changes that result in a stress response generally due to an increase in tissue oxygen demands—a patients' ability to withstand this stress depends primarily on their cardiorespiratory fitness (Barakat et al., 2016). Further, prolonged periods of physical inactivity in the post-operative phase induce a loss of muscle mass, cardiopulmonary deconditioning, pulmonary complications, and psychological distress (Pouwels et al., 2016), all of which can be offset by enhanced fitness. Pre-surgical exercise training therefore represents an encouraging means of improving surgical outcome (Weston et al., 2016) and is potentially beneficial for patients with abdominal aortic aneurysm disease (Pouwels et al., 2015).

Abdominal aortic aneurysm (AAA) is a frequently lethal disease (Lederle et al., 2000). It is generally an asymptomatic condition until aneurysm rupture occurs, precipitating sudden collapse or death (Waton et al., 2013). Due to the high mortality associated with emergency surgery, elective repair is the preferred option when AAA size breeches 5.5 cm and surgical outcome can be influenced by a patient's pre-operative cardiorespiratory fitness (Prentis et al., 2012; Grant et al., 2015). Fortunately, fitness is a modifiable factor during the pre-operative phase—if there is a cause and effect relationship with the post-operative course, patients undergoing major abdominal and thoracic surgery will benefit from pre-operative interventions to improve their fitness (Hoogeboom et al., 2014).

Patients with large AAA disease (>5.5 cm) should undergo elective surgical intervention within 8 weeks of referral (The Vascular Society, 2011); yet following initial consultation, often only 4–5 weeks remain before surgery. As such, exercise programmes incorporated into the pre-operative pathway need to be effective and time-efficient (Tew et al., 2014; Weston et al., 2016). Consequently, high-intensity interval training (HIT) represents an attractive strategy for the improvement of pre-surgical fitness in AAA patients (Jack et al., 2011) given that rapid fitness gains are possible in a short period of time (Weston M. et al., 2014). As yet though, exercise interventions undertaken by AAA patients have been confined to moderate exercise intensities (Kothmann et al., 2009; Tew et al., 2012; Myers et al., 2013) and the feasibility of HIT in AAA patients remains unknown.

The HIT-AAA project (Tew et al., 2014)—a multi-center feasibility study on the efficacy of a pre-operative HIT intervention on post-operative outcomes in patients undergoing elective AAA repair—therefore represents the first attempt to exercise AAA patients to high intensities. Central to the internal validity of all intervention trials is intervention fidelity, which refers to the extent an experimental manipulation has been implemented as intended, in a comparable manner to all participants (Resnick et al., 2011; Taylor et al., 2015). In the context of exercise trials, an assessment of fidelity permits an understanding of whether the exercise was performed at the prescribed intensities, at all study sites and throughout all phases of the study. As such, our aim here was to present a detailed appraisal of exercise data collected during the HIT-AAA trial. While we have reported a full description of the HIT protocol and exercise session responses, our evaluation is confined to an examination of the exercise undertaken in the HIT-AAA intervention and not the intervention effects. Data on other aspects of feasibility (e.g., rates of recruitment and retention) and intervention effects will be reported elsewhere.

## Methods

### Experimental design

The HIT-AAA project was a three-site, two-arm, parallel-group, randomized controlled feasibility study (trial registration ISRCTN09433624) approved by the North East-Tyne & Wear South Research Ethics Committee (13/NE/0116). Study recruitment was undertaken from August 2013 to December 2015 and all participants provided written informed consent. The HIT intervention was performed in the 4 weeks preceding surgery and the exercise protocol was based on prior HIT programmes shown to be safe and effective for improving cardiopulmonary fitness in patients with heart failure (Wisløff et al., 2007) and coronary heart disease (Rognmo et al., 2012). While the study's protocol, inclusion and exclusion criteria have been published elsewhere (Tew et al., 2014), for the purpose of this paper the methods pertaining to the HIT intervention are described below.

### Participants

A total of 27 (two female) participants (mean ± SD age: 74.3 ± 5.7 years, height: 172.3 ± 9.0 cm, body mass: 79.1 ± 15.9 kg, aneurysm size: 6.0 ± 0.4 cm) were randomized to the HIT intervention. Following study enrolment, all participants underwent a baseline cardiopulmonary exercise test (CPET) on a cycle ergometer. The mean baseline anaerobic threshold (AT) and peak oxygen consumption (VO_2_peak) were 11.0 ± 2.1 mL/kg/min and 16.5 ± 3.7 mL/kg/min, respectively. Mean recorded baseline power output on the CPET was 54 ± 19 watts at AT and 98 ± 29 watts at VO_2_peak.

### Exercise intensity: prescription and measurement

During the first HIT session, all participants exercised to the power output observed at the AT determined on the baseline CPET. In subsequent sessions, power output was increased until the patient reported ratings of perceived exertion (RPE) of five (“hard”) to seven (“very hard”) on Borg's CR-10 scale (Borg, 1982) at the end of each interval. The precision of RPE data during exercise can, however, be enhanced by differentiating perceptual reports according to their specific mediators with local and central being regarded as the most important signals (Borg et al., 2010). As such, we collected separate (differential) RPE scores for the perceived exertion in the legs (RPE-L) and the perceived sense of breathlessness in the chest (RPE-C). Each patient was familiarized with the scale and the recommended researcher instructions for scale administration were used (Borg, 1982). The research nurse and physiotherapist supervising each HIT session recorded power output (watts), blood pressure (manually via sphygmomanometer) and RPE at the end of each interval. Heart rate data were recorded continuously at 5-s intervals throughout the entire exercise session (Polar RS400, Kempele, Finland) with data download procedures as per Taylor et al. (2015).

### HIT protocol

Participants completed three HIT sessions per week throughout the 4-week training period with 48 h recovery in between sessions (e.g., Monday, Wednesday, Friday). All exercise sessions were hospital based, supervised by a physiotherapist and research nurse and performed on a cycle ergometer (Optibike Med, Ergoline, Germany), with sessions performed >3 h after waking given the higher frequency of cardiovascular events during the morning hours (Thompson et al., 2007). Each HIT exercise session commenced and finished with a 10-min warm up and 5-min cool down of unloaded cycling. For HIT sessions one to three, all participants performed 8 × 2 min repetitions, with each interval interspersed with a 2-min period of active (unloaded cycling) or passive (rest) recovery. Following this, for HIT sessions 4–12 participants had the choice of either the 2-min protocol or a 4-min protocol which consisted of 4 × 4 min repetitions with each interval interspersed with a 4-min period of active (unloaded cycling) or passive (rest) recovery. Where surgery was delayed beyond week five, participants entered a maintenance phase of training consisting of one HIT session per week. Here, the participants had a choice of which HIT protocol to undertake, either 8 × 2 min or 4 × 4 min.

### Exercise safety

Power output in any exercise session was maintained or reduced (termed workload reduction) if systolic blood pressure (SBP) exceeded 180 mm Hg (Isselbacher, 2005) or if heart rate exceeded 95% of the maximum observed on baseline CPET.

### Study compliance

In our protocol, a participant was deemed compliant if they completed ≥75% of the scheduled sessions, that is, at least 9/12 sessions for the 4-week intervention, plus all once-weekly maintenance sessions if surgery was delayed (Tew et al., 2014). Across the 4-week intervention period, 20 out of 27 participants attended a minimum of 75% of the scheduled intervention sessions. Fifteen of the 27 participants entered the maintenance phase of the study, with 36 out of a total of 40 (90%) prescribed sessions attended. The mean number of maintenance sessions offered per participant was 1 (range 0–9).

**Figure 1:**
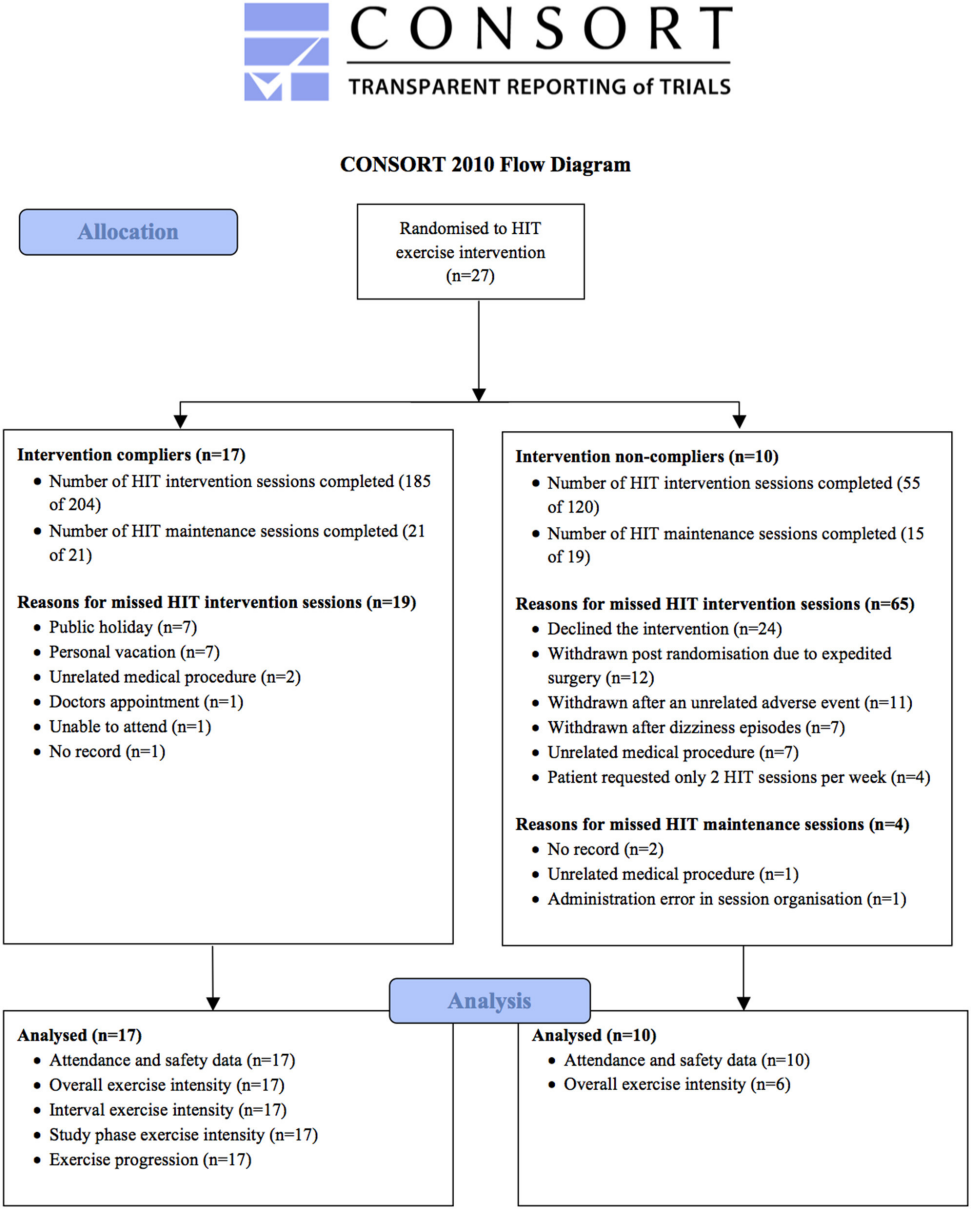
**CONSORT flow chart**.

Overall, a total of 17/27 participants (63%) met our compliance criteria. Of this cohort, the mean number of HIT sessions attended (per participant) during the 4-week intervention and subsequent maintenance phases was 11 (range 9–12) and 1 (range 0–4), respectively. By comparison, the 10 non-compliant participants attended a mean (range) of 6 (0–12) HIT sessions during the 4-week intervention and 2 (0–9) during the maintenance phase. Our CONSORT diagram (Figure 1) summarizes participant flow, reasons for all missed HIT sessions and number of participants included in the fidelity analysis for compliant and non-compliant groups. Given the relatively low number of HIT sessions performed by the non-compliant participants, our in-depth analysis of the exercise data is confined to the 17 compliant participants.

### Statistical analysis

Differences in baseline fitness variables between compliant and non-compliant participants were examined using an unpaired *t*-test, with magnitude based inferences subsequently applied (Hopkins, 2007). Here, inferences were based on standardized thresholds for small, moderate and large differences of 0.2, 0.6, and 1.2 of the pooled between-subject standard deviations (Hopkins et al., 2009). The chance of the difference being substantial or trivial was interpreted using the following scale: 25–75%, possibly; 75–95%, likely; 95–99.5%, very likely; >99.5%, most likely (Batterham and Hopkins, 2006). Descriptive statistics were used to calculate the session attendance data. The difference in the proportion of workload reductions between compliant and non-complaint participants was calculated, with uncertainty in the estimates expressed as 90% Confidence Limits (CL). We determined the proportion of HIT repetitions that met our prespecified RPE criteria for high-intensity, along with the median and interquartile range (IQR) for these proportions. Linear mixed modeling (SPSS v.23, Armonk, NY: IBM Corp) was used to calculate the effect of compliance (compliant, non-compliant), interval duration (2 min, 4 min) and training phase (intervention, maintenance) on our measures of exercise intensity (power, RPE-L, RPE-C, heart rate) and also to examine the difference between differential RPE scores (RPE-L, RPE-C), with magnitude-based inferences subsequently applied. For all variables, we classified the magnitude of effects mechanistically, whereby if the 90% confidence limits overlapped the thresholds for the smallest worthwhile positive and negative effects the effect was deemed unclear (Hopkins et al., 2009). The within-subject variability (expressed as a standard deviation [SD]) in RPE-L and RPE-C was determined via a linear mixed model with a random intercept, with the SD doubled to interpret its magnitude (Smith and Hopkins, 2012). For the assessment of exercise progression in power, RPE-L, RPE-C, and heart rate across the 4-week HIT intervention we applied a linear mixed model. “Session” (1–12) was entered as fixed effect, with a random slope and intercept for session (unstructured covariance matrix). We used the slope of the relationship between session and outcome to derive the percentage change (power output) or raw change (RPE, heart rate) over the 4-week intervention.

## Results

### Participants

Demographic and baseline fitness levels for all 27 patients randomized to the HIT-AAA exercise intervention are displayed in Table 1. When compared to non-compliers, study compliers had a higher baseline AT, baseline VO_2_peak and power output recorded at AT and VO_2_peak with the magnitude of all effects being likely small (possibly moderate). The between-group differences in age, height, body mass and aneurysm size were unclear.

**Table 1:** **Demographic and descriptive variables at baseline for HIT-AAA compliers (***n*** = 17) and non-compliers (***n*** = 10)**.

	**Compliers**	**Non-compliers**	**SWC**	**Difference; ±90% CL**
Age (years)	74.8 ± 5.5	73.3 ± 6.0	1.1	1.5; ± 4.5
Height (cm)	171.7 ± 8.7	173.2 ± 8.9	1.8	−1.5; ± 7.0
Body mass (kg)	79.1 ± 11.4	79.2 ± 21.7	3.5	0.1; ± 13
Sex (male/female)	16/1	9/1	−	−
Aneurysm size (cm)	6.0 ± 0.5	6.1 ± 0.3	0.1	−0.1; ± 0.3
AT (mL/kg/min)	11.4 ± 2.1	10.1 ± 1.8	0.4	1.3; ± 1.6
Power output at AT (watts)	58 ± 18	46 ± 18	3.6	12; ± 15
VO_2_peak (mL/kg/min)	17.5 ± 3.6	14.6 ± 3.0	0.7	2.9; ± 2.8
Power output at VO_2_peak (watts)	105 ± 29	83 ± 21	5.1	22; ± 22

SWC, threshold value for the smallest worthwhile change (0.2^*^pooled between-subject standard deviation); CL, confidence limits; AT, anaerobic threshold; VO_2_ peak, peak oxygen consumption.

### HIT attendance and safety

Attendance and safety data for the exercise sessions are presented in Table 2. Overall session attendance was 74% for the 4-week intervention and 95% for the maintenance phase of the study (see Figure 1). Attendance was higher in the compliant participants and there were clear patient preferences for 2-min intervals. Thirteen (of 17) compliant and seven (of 10) non-compliant patients experienced workload reductions in-line with our safety criteria. The difference in the proportion of workload reductions between compliant and non-compliant participants was 7.5% (±90% Confidence Limits 2.5%). One participant (a complier) experienced an adverse event (angina episode) requiring the use of Glyceryl trinitrate spray 29 min into exercise session eight (session completed). This participant missed session nine due to cardiology review, but returned to complete the remaining three exercise sessions plus one maintenance session. One participant (a non complier) was investigated for prodromal symptoms of dizziness during exercise. Subsequent cardiac evaluation was normal but the participant was not able to resume training due to the time taken for referral.

**Table 2:** **Exercise attendance and safety data for HIT-AAA compliers (***n*** = 17) and non-compliers (***n*** = 10)**.

	**Compliers**	**Non-compliers**
Mean intervention session attendance (%) (range)	91 ± 9% (75–100%)	46 ± 42% (0–100%)
Mean maintenance session attendance (%) (range)	100% (0%)	81% ± 14% (67-100%)
Total number of HIT repetitions performed	1410	396
Number of 2-min intervals	1172 (83%)	240 (61%)
performed		
Number of 4-min intervals	238 (17%)	156 (39%)
performed		
Total number of workload reductions	36 (2.6%)	40 (10.1%)
Number of workload reductions	19 (1.6%)	31 (12.9%)
for 2-min intervals		
Number of workload reductions	17 (7.1%)	9 (5.8%)
for 4-min intervals		
Number of adverse events	1	0

*HIT, high-intensity interval training*.

### Exercise intensity

Mean exercise intensity data are presented in Table 3. When compared to non-compliers, power output (magnitude of effect—likely small/ possibly moderate), RPE-C (likely small) and RPE-L (possibly small) were higher for the compliers. The differences in heart rate and systolic blood pressure were unclear. For the study compliers, the percentage of HIT repetitions meeting the compliance criteria for high-intensity training was 30% (IQR 16%, 68%) for RPE-L and 16% (10%, 42%) for RPE-C. Analysis of the RPE scores revealed a most likely small difference (0.6 Arbitrary Units [AU]; 90% confidence limits ±0.1 AU) between RPE-L and RPE-C. The magnitude of the within-subject variability was large for both RPE-L and RPE-C.

**Table 3:** **Exercise intensity data, within-participant variability and inferential statistics for the difference between compliers (***n*** = 17) and non-compliers (***n*** = 6)**.

	**Compliers mean ± SD; within-subject variability**	**Non-compliers mean ± SD; within-subject variability**	**SWC**	**Difference; ±90% CL**
Power output (watts)	69.1 ± 16.4;	56.2 ± 16.5;	3.3	13; ± 13
	8.9	7.4		
RPE-L (au)	4.1 ± 2.0;	3.4 ± 1.4;	0.3	0.7; ± 1.1
	1.4	1.0		
RPE-C (au)	3.5 ± 1.9;	2.8 ± 1.1;	0.2	0.7; ± 0.9
	1.6	1.0		
Heart rate (% of maximal)	81.7 ± 8.5;	83.3 ± 9.0;	1.7	−1.6; ± 7.3
	5.7	3.6		
SBP (mm Hg)	159 ± 17;	154 ± 26;	3.3	4.6; ± 13.4
	12.2	13.0		

SD, standard deviation; SWC, threshold value for the smallest worthwhile change (0.2^*^pooled between-subject standard deviation); CL, confidence limits; RPE-L, leg ratings of perceived exertion; RPE-C, chest ratings of perceived exertion; SBP, systolic blood pressure.

### Interval duration and study phase

The effect of interval duration (Table 4) on the exercise intensity of the compliant participants was higher RPE-L (likely small) and RPE-C (possibly small) for the 2-min intervals and higher SBP (very likely small) and heart rates (likely small) on the 4-min intervals. The effect on power output was trivial. Also presented in Table 4 is the effect of study phase on exercise intensity. Here, RPE-L (very likely small), RPE-C and heart rate (both likely small) were higher during the 4-week intervention phase of the study, with power output higher (likely small) during the study's maintenance phase. The effect on SBP was trivial.

**Table 4:** **Exercise intensity data for interval duration (2-, 4-min intervals) and study phase (intervention, maintenance) in the HIT-AAA compliers (***n*** = 17)**.

	**2-min intervals**	**4-min intervals**	**SWC**	**Difference; ±90% CL**
**INTERVAL DURATION**				
Power output (watts)	68.5 ± 17.6	71.3 ± 12.2	3.0	2.8; ± 1.5
RPE-L (au)	4.2 ± 1.5	3.6 ± 1.1	0.3	−0.6; ± 0.2
RPE-C (au)	3.6 ± 1.1	3.1 ± 0.8	0.2	−0.5; ± 0.3
Heart rate (% of maximal)	81.1 ± 6.7	83.5 ± 4.8	1.2	2.5; ± 1.0
SBP (mm Hg)	158 ± 12	163 ± 8	2.1	5.6; ± 2.1
	**Intervention**	**Maintenance**	**SWC**	**Difference; ± 90% CL**
**STUDY PHASE**				
Power output (watts)	68.6 ± 17.5	74.1 ± 14.3	3.2	5.5; ± 1.4
RPE-L (au)	4.1 ± 1.5	3.4 ± 1.2	0.3	−0.7; ± 0.2
RPE-C (au)	3.6 ± 1.1	3.0 ± 1.0	0.2	−0.6; ± 0.3
Heart rate (% of maximal)	81.9 ± 6.5	79.8 ± 5.5	1.2	−2.2; ± 0.9
SBP (mm Hg)	159 ± 13	156 ± 11	2.4	−2.6; ± 2.0

SD, standard deviation; SWC, threshold value for the smallest worthwhile change (0.2^*^pooled between-subject standard deviation); CL, confidence limits; RPE-L, leg ratings of perceived exertion; RPE-C, chest ratings of perceived exertion; SBP, systolic blood pressure.

### Exercise progression

**Figure 2:**
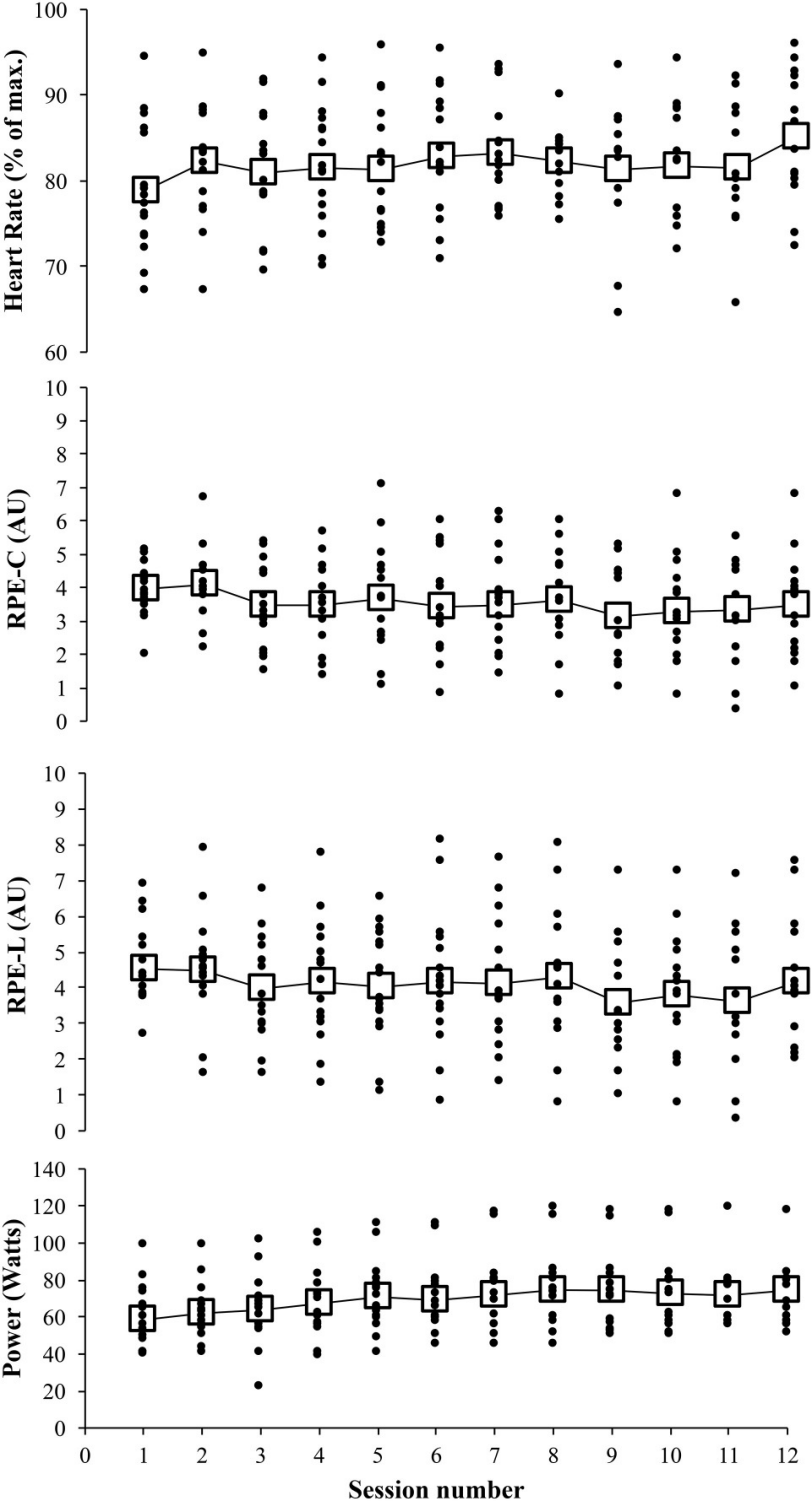
**Mean (large open squares) and individual (small closed circles) power output, leg ratings of perceived exertion (RPE-L), breathless/chest ratings of perceived exertion (RPE-C), and heart rates exercise across the HIT intervention (sessions 1–12)**.

Presented in Figure 2 are the mean exercise intensity scores per HIT session along with the individual data points (compliers only) to illustrate the variability around the mean score. The regression slope revealed an increment of 23% (90% Confidence Limits ±12.5%) in power output across the 12 HIT sessions with a between-subject variability of ±23% (±10%). The rate of change in RPE-L and RPE-C across the 12 HIT sessions was −0.7 AU (±0.5 AU) and −0.8 (±0.7 AU), respectively with a between-subject variability of 1.0 AU (±0.4 AU) and 1.3 AU (±0.5 AU). There was an increment in heart rate of 3.1 percentage points (±2.9 percentage points) with a between-subject variability of 5.4 percentage points (±2.6 percentage points).

## Discussion

Pre-operative exercise training represents a plausible means of improving surgical outcome. While high-intensity interval training has promise for the effective and time-efficient enhancement of pre-surgery fitness (Weston et al., 2016), it is not yet known whether patients with large abdominal aortic aneurysms can exercise at a high intensity while remaining within safe blood pressure and heart rate limits. Using the HIT-AAA trial data, our aim here was to undertake a detailed evaluation of the exercise sessions. When adhering to pre-determined safety criteria, our results show primarily the low fidelity of the HIT programme—a consequence of attendance and also non-compliance due to an inconsistent and lower than prescribed exercise intensity. Our data do, however, show that not only is it possible to exercise this high-risk patient population at moderate to hard intensities, the progression of cycling power output over the duration of a short-term training programme is also achievable.

### HIT attendance, compliance, and safety

Our overall session attendance for the 27 participants across the 4-week HIT intervention compares favorably with an attendance range of 58–77% for older adults undertaking exercise programmes (Picorelli et al., 2014). The figure is, however, considerably below the 94% reported by Tew et al. (2012) for an endurance exercise intervention performed by patients with small AAA disease in a surveillance programme. Given that greater cardiorespiratory endurance (Rhodes et al., 1999) and better physical function (Picorelli et al., 2014) are associated with better adherence, such discrepant findings may well be explained by our participants being older, having larger aneurysms and lower cardiorespiratory fitness. As expected, there were clear differences in the session attendance rates between compliant and non-compliant participants, which again could likely be a consequence of fitness given that our compliant participants had substantially higher cardiorespiratory fitness levels. Further, the higher level of cardiorespiratory fitness and ability to cycle to higher power outputs explains why our complaint participants were able to exercise at higher absolute and relative exercise intensities throughout the study and possibly why they recorded fewer safety workload reductions. It has been previously reported from meta-analyses that HIT favors the less fit (Weston M. et al., 2014; Milanović et al., 2015) which poses an interesting juxtaposition as lower fitness in the present study was associated with non-compliance; although participant fitness and health in the present study were substantially lower than that reported in the afore-mentioned meta-analyses.

Evaluations of fidelity in exercise training interventions should ideally address session attendance *and* compliance (meeting the prescribed exercise intensity), as this interaction constitutes the dose of the intervention and influences the physiological response to exercise training (Taylor et al., 2015). Furthermore, while it is difficult to classify intervention fidelity, Taylor et al. (2015) felt that high-intensity criterion attainment in a little over half of the HIT repetitions per participant, represented moderate intervention fidelity. With this in mind, the overall fidelity of our exercise intervention is probably best described as low, given the poor compliance to our predetermined RPE criteria for high-intensity (~23%) and the overall intensity of the HIT repetitions being lower than prescribed. Indeed, our mean RPE scores fell in the range of moderate to somewhat hard on the CR10 scale, when the lower limit of our target was hard. Although patient medication (e.g., beta blockers) blunt the exercise heart rate response, our training heart rates support the moderate to hard intensity of the exercise intervention as a mean peak heart rate of 82.2% is slightly below that required for HIT (e.g., 85–95% of peak heart rate; Weston K. S. et al., 2014).

While mean exercise session data provide valuable information, the values do not quantify the degree of consistency in exercise intensity across the intervention (Taylor et al., 2015). In the present study, the observed magnitude of the within-subject variability in RPE was large. Such variability illustrates an inconsistent exercise dose across the intervention as it encompasses a vista of intensities from easy through to hard (Borg, 1982). For example, when the within-subject variability for RPE-C (1.6 AU) is added to and subtracted from the mean of 3.5 AU, the estimate for RPE-C typically varies from easy (1.9 AU) to hard (5.1 AU). Such variability further supports low fidelity as the exercise dose was not applied consistently across the intervention.

The success of our prescribed exercise intensity contrasts with previous exercise training studies undertaken on AAA patients. Tew et al. (2012) reported on a thrice weekly, 12-week exercise programme targeted to fall within the range of 12 to 14 on the Borg 6–20 scale. The authors concluded that moderate-intensity endurance exercise training (mean RPE, 11.8 ± 0.8 AU, mean heart rate, 72 ± 8% of age-predicted maximum heart rate) is feasible in patients with small abdominal aortic aneurysms (mean aneurysm size 4.1 ± 0.1 cm). In another study examining the effects of exercise training in AAA patients (aneurysm size 3.0–5.0 cm), Myers et al. (2013) reported a mean training intensity of 98 ± 7% of target heart rate, which was initially 60% of heart rate reserve, increasing to 80%, with perceived exertion was targeted to fall within the range of 12–14 on the Borg 6–20 scale. Kothmann et al. (2009) also used a moderate-intensity exercise dose (12–14 on the Borg scale) in their pilot study examining the effect of short-term exercise training on aerobic fitness in AAA patients (aneurysm size 3.0–5.1 cm); however, the authors did not report any exercise training data. While a full comparison of between-study exercise intensity is not possible given substantial inconsistency in the detail of exercise data presented, clear differences in prescribed exercise intensity, namely moderate versus high-intensity, are apparent, yet the reported intensities appear similar (e.g., a moderate to somewhat hard intensity).

Of interest in the Kothmann et al. (2009) study was that the authors prescribed moderate continuous exercise rather than high-intensity interval training for “safety reasons.” The safety concerns surrounding AAA patients when exercising are combined excessive rises in SBP and heart rate causing aneurysm rupture, which is a physiologically catastrophic insult carrying a mortality of about 75% (The Vascular Society, 2011). Precise exercise training safety criteria are, however, lacking from previous AAA patient exercise training studies (Kothmann et al., 2009; Tew et al., 2012; Myers et al., 2013). Furthermore, aneurysm size in these studies was small (~40 mm), whereas we recruited patients with large abdominal aortic aneurysms (55–70 mm diameter), which reinforced the need for our exercise training safety guidelines as SBP should not rise above 180 mm Hg for AAA patients wishing to engaging in vigorous aerobic exercise (e.g., running or cycling; Isselbacher, 2005; Myers et al., 2012).

There were no serious adverse events. There was one adverse event in one of the 17 compliant participants in a total of 55 person-hours of exercise—an event rate of 1.8 per 100 person-hours. The 95% simple Bayesian confidence interval (Ludbrook and Lew, 2009) for this rate is 0.4–9.6 events. The upper limit of the confidence interval may be viewed as an estimate of the maximum risk consistent with the data (Batterham et al., 2014); the chance of an adverse event could be as high as around one in every 10 person-hours. However, proportions or rates derived from small integer counts in small samples are unstable, as a small change in numerator can affect the estimates substantially. For example, if we had observed no adverse events in our study the upper confidence limit would be 6.4 events/100 person-hours, versus 14.9/100 person-hours with three adverse events. Clearly, much more data is required to evaluate the safety of the intervention properly in this patient group.

### Interval duration

We found substantially higher RPE scores (~0.5 AU) following 2-min intervals when compared to 4-min intervals despite trivial or small differences in power output during the study intervention and maintenance phases, respectively. Shorter intervals have recently been perceived as requiring less effort, with frequent breaks from severe intensities possibly contributing to the lower perceived effort (Kilpatrick et al., 2015). In contrast, other authors have reported either no difference in RPE between 1- and 4-min intervals despite power output being significantly higher during the shorter intervals (Tucker et al., 2015) or that the RPE of interval training programmes is more strongly related to work intensity than accumulated duration (Green et al., 2009). Heterogeneous study populations with a variety of exercise experience (e.g., patients, obese/overweight, active) may contribute to these disparate findings. Further research on the interplay of interval duration, power output (or treadmill speed) and RPE in patient populations is therefore recommended. This recommendation is further emphasized by the juxtaposition of recent HIT research whereby shorter intervals are more palatable than longer intervals for novice exercisers (Kilpatrick et al., 2015), yet there is an increased adaptive response on VO_2max_ following longer intervals (Milanović et al., 2015).

### Exercise progression

Our primary focus has centered on exercise intensity, yet a detailed collection and analysis of exercise training data permits the objective appraisal of success in other aspects of a training study, namely exercise progression. To facilitate a positive adaptation to training, the prescription of exercise needs to advance over time. Very often training intensities are re-defined and re-prescribed following a mid-programme assessment of exercise capacity (West et al., 2015); however, this is at greater fiscal cost and the assessments can disrupt the training programme (Weston et al., 2016). In the present study we used relative measures of exercise intensity, as RPE scores provide a practical and valid means of ensuring training progression is inherent within programmes (Weston et al., 2016). Despite our exercise data showing a lower than prescribed exercise intensity, a substantial increase in power output across the 12 HIT sessions demonstrates clear exercise progression. We do, however, acknowledge that there was substantial between-participant variability in this estimate. While it is difficult to reconcile the magnitude of this progression with other clinical exercise trials—previous work largely focuses on training outcome as opposed to process—our data are higher than that reported for young sedentary subjects (18%) (Foster et al., 2015) and elite athletes (4.6%) (Purkhús et al., 2016). We also found a reduction in RPE across the duration of the intervention, a finding consistent with recent successful HIT programmes in healthy middle-aged men (Saanijoki et al., 2015), sedentary (Astorino et al., 2016) and overweight women (Smith-Ryan et al., 2016). Of these studies, however, only Saanijoki et al. (2015) quantified exercise progression over the same time period during which RPE decreased.

As heart rate can be an unreliable measure of exercise intensity in patients on medication, we used differential RPE scores to enhance the sensitivity of the exercise data we collected. Consistent with previous studies, we found peripheral ratings of exertion to be higher than central ratings (Pandolf et al., 1975; Green et al., 2009; Borg et al., 2010; McLaren et al., 2016). The magnitude of the difference in the two perceptual responses was small, which is consistent with recent reports in athletic populations (McLaren et al., 2016). This finding helps to explain the lower compliance for RPE-C as our high-intensity criterion was the same for both measures of RPE. Previously, Pandolf et al. (1975) reported substantially lower RPE-L following a 6-week period of cycling training, yet no reduction in RPE-C. The authors concluded that local factors dominate the exertional perception when cycling and that focus on reporting these sensations could interfere with the perception of central factors. In the present study, however, the magnitude of reduction in RPE-L and RPE-C over the duration of the study was consistent (e.g., −0.7 AU), suggesting the absence of any such reporting inference and showing that differential RPE do indeed offer a sensitive evaluation of exercise intensity.

### Limitations and clinical applications

A major limitation of the present study was that we were unable to exercise our participants to the prescribed exercise intensity, namely high intensity. We cannot, however, rule out the possibility of underreported RPE scores due to the influence of observer sex. Males report lower RPE values when a female observer, as opposed to male, is in the room (Winchester et al., 2012) and all but one of our participants were male and at each site RPE data were collected by female nurses and physiotherapists. As such, Halperin et al. (2015) recently recommended that the sex and number of observers should be standardized to limit the effects of this potentially confounding variable. We also acknowledge that less stringent safety criteria, especially for SBP, could have enabled patients to exercise at higher intensities. Nonetheless, as this was the first study attempting to exercise this high-risk patient population to high intensities, it was important that our safety criteria, and also the extent to which the patients were pushed during exercise, reflected a conservative approach. Given that pre-operative exercise therapy exerts beneficial effects on physical fitness and post-operative outcome measures (Pouwels et al., 2016), our data have clear clinical application by showing it is possible to progressively exercise this high-risk population at moderate to hard intensities. Finally, an in-depth assessment of fidelity in multi-center exercise interventions should examine the consistency of the exercise dose across the different sites, yet given the relatively low sample size for each of our three study sites we elected not to include between-site comparisons.

## Conclusion

This is the first study to provide a detailed quantification of the exercise sessions performed across a HIT intervention in patients awaiting AAA repair. With an adherence to stringent exercise safety criteria for blood pressure and heart rate, our results showed an inconsistent and lower than prescribed exercise intensity. Although the attainment of a high-intensity training stimulus in this patient population may be difficult, our data do show it is possible to exercise this high risk patient population at moderate to hard exercise intensities.

## Author contributions

All authors contributed equally to the study design and were involved in the data collection, conceptualization and drafting of the article. MW and AMB completed the statistical analysis. All authors contributed to the writing of the manuscript and approved the final version of the manuscript.

## Funding

This paper presents independent research funded by the National Institute for Health Research (NIHR) under its Research for Patient Benefit (RfPB) Programme (Grant Reference Number PB-PG-1111-26068). The views expressed are those of the authors and not necessarily those of the NHS, the NIHR or the Department of Health.

### Conflict of interest statement

The authors declare that the research was conducted in the absence of any commercial or financial relationships that could be construed as a potential conflict of interest.
